# The contribution of peer research in evaluating complex public health interventions: examples from two UK community empowerment projects

**DOI:** 10.1186/s12889-022-14465-2

**Published:** 2022-11-24

**Authors:** Kris Southby, Susan Coan, Sara Rushworth, Jane South, Anne-Marie Bagnall, Tiffany Lam, Jenny Woodward, Danial Button

**Affiliations:** 1 Leeds Beckett University, 519 Portland, City Campus, Leeds, LS1 3HE UK; 2 Sustrans UK, 2 Cathedral Square, Bristol, BS1 5DD UK; 3 New Economics Foundation, 10 Salamanca Place, London, SE1 7HB UK

**Keywords:** Peer research, Evaluation, Research methods, Complex interventions, Community empowerment

## Abstract

**Background:**

Peer-research is steered and conducted by people with lived experience of the issues being researched. This paper explores the value of peer-research in two complex public health intervention evaluations in the UK.

**Methods:**

Reports from 18 peer research projects, completed by residents from 12 communities in the UK taking part in two community empowerment interventions, were analysed using cross-case analysis.

**Results:**

Undertaking peer research helped to build the evaluation and research skills within individual projects as well as providing data on other outcomes related to the programmes Theory of Change. Some peer researchers, however, felt unprepared for the activity despite support from the academic team and were unsatisfied with project outcomes. While peer research projects provided more opportunities for local residents to engage with the overall evaluations, there was an overreliance on people closely connected to the programmes to be peer researchers. The peer research projects explored topics that were broader than the aims and objectives of the overall programme evaluations. All provided insight into the context in which projects occurred, while some also informed understanding of programme change mechanisms.

**Conclusions:**

Including peer research as part of complex public health intervention evaluations can help uncover important contextual and ecological details beyond the reach of more traditional evaluation data collection. Peer research can also empower and build research/evaluation capacity within communities, which is particularly pertinent for community empowerment interventions.

## Introduction

Evaluation of complex public health interventions – those that have multiple interacting components and non-linear causal pathways [1] – requires a multidisciplinary approach [2, 3], triangulation between the insights of different methodologies [4], and consideration of the context and ecology in which interventions occur [5]. Methodologies, like ethnography [6], photo-elicitation [4], and in-depth longitudinal case studies [7], have emerged as means to capture complexity in public health intervention evaluations that would have otherwise been overlooked.

This paper supports the need for high quality, robust, realistic and proportionate evaluation methodologies [8] by examining the contribution of peer research in two community empowerment intervention evaluations in the UK: Local People (LP) and Local Conversations (LC). Community empowerment approaches are complex public health interventions because of the multiple, usually non-standardised, components involved and multiple layers of interaction within the intervention and with the local ecology [6, 9, 10]. The LP and LC programmes involved residents in fifty disadvantaged neighbourhoods being supported by local and national voluntary and community sector (VCS) organisations to come together to increase their control and influence over the things that matter to them locally to improve social determinants of health, local services, and health and wellbeing, ultimately contributing to reduced health inequalities. (See the programme website for more detail [11]).

Peer research is steered and conducted by people with lived experience of the issue being studied, who adopt the role of active researchers to collect data from their peers about their experiences. It is a way to reach into communities and include seldom heard voices – which is particularly useful when tackling health inequalities [12] – establishing rapport with participants, empowering and upskilling participants and communities [13, 14], and exploring issues that participants are less willing to raise with academic researchers [15]. There are apparent synergies between peer research and community empowerment: involvement in peer research is an opportunity for community members to assume control, to learn new skills, and increase their capacity [4, 16, 17], which, in turn, can support the sustainability of community-based interventions [16]. However, concerns with the method include maintaining confidentiality and data quality [18], being time-consuming and unpredictable [19], and getting the balance right between empowering participants and ensuring academic rigour [20]. The emergence of ‘lay experts’ and ‘expert patients’ indicates the increasing value of experiential knowledge in health and public health fields [21], and peer research is increasingly acknowledged by governments and commissioners [22]. Peer research fits within a wider group of community-based participatory research methodologies that attempt to reframe research with a health equity perspective [16]. However, the extent of peer research’s contribution to evaluating complex public health interventions is currently underexplored. The value of the broader class of “community-based participatory research” to intervention research [16] and of peer research as a part of multi-modal evaluation design to account for complexity [7] has been interrogated. This paper describes the specific methodology and presents an analysis of the contribution of peer research to the evaluation of the LP and LC complex public health interventions. In this context, peer research projects were delivered across a wide variety of neighbourhoods and communities experiencing socioeconomic disadvantage.

## Methodology

In order to explore the contribution of peer research, a secondary analysis of peer research reports produced as part of the LC and LP projects was undertaken using cross-case analysis [23]. A matrix was used to extract key information from, and facilitate comparison between, each report. Specific reports are referenced here by either LC or LP and a number (1–5) denoting which project it came from and a number (1–3) denoting the round of data collection.

In total, 18 peer research projects were completed (see Table 1) by twelve different LC and LP projects. Peer research took place during each of the three data collection phases in LC (2017, 2019, 2020–21) but only once in LP (2018). Peer researchers’ reflections on the process of undertaking peer research were captured in each project report. The staged peer research process is described in Table 2. Two enforced changes to the planned process due to Covid-19 were: 1) training and support moved from face-to-face to online delivery; and 2) co-analysis workshops between peer researchers and supporting evaluation team members were replaced with analysis being done by supporting evaluation team members only and corroborated by peer researchers via email. Peer researchers were not involved in producing this paper. Whilst this is contrary to the principles of peer research, there appeared to be little appetite among peer researchers to be involved when we began the writing process.

**Table 1:** Summary of completed peer research projects

Project	Round	Topic	Method(s)	Sampling	Peer researchers	Participants	Main findings	ToC component
LC1	1	Young resident’s experience of the area	Photovoice	n/a	7 (young people)	7	Local identify & culture; close-knit families; lack of space; poor-quality housing; fly-tipping; social divisions based on language; places of unity; communal celebrations.	Context
LC2	1	Female resident’s experience of the area	Semi-structured interviews	Convenience	8 (local women)	16 (8 ‘older’, 8 ‘younger’)	Range of skills/interests/aspirations; common family pressure; improving situation for women; mental health stigma; low mental health literacy.	Context
LC3	1	Residents’ views of estate regeneration; community activity involvement	Survey	Convenience	4	55	Residents feel marginalised/ignored; improve current homes; fears of tenancy changes; dislike private housing; strong sense of belonging but low trust; fear of crime.	Context
LC4	1	Local experience of asset transfer	Semi-structured interviews	Convenience	6	25 (managers, users, non-users)	Community assets important to physical and emotional wellbeing; Assets ‘dumped’ on community; necessary skills/knowledge in place; CAs to be more tailored to community; fear of future viability.	Context; mechanism of change
LC5	2	Value of local foodbank project; community activity involvement	Survey	Convenience	4	26	Information about service users; service highly valued; potential improvements; want to be involved in community activity; need to match people skills with roles.	Context; mechanism of change
LC2	2	Intergenerational relations; making community events more inclusive	Semi-structured interviews	Convenience	9	25 (14 female, 11 male)	Poor intergenerational relationships; negative view of teenagers; desire for community events; using complimentary skills to bring people together.	Context; mechanism of change
LC3	2	Community activity involvement	Mixed-methods – survey + semi-structured interviews	Convenience	5	5 interviews 42 surveys	Broad support for LC project; involving more people is good; multiple barriers to taking part (e.g. childcare, work); limited want to be more involved.	Context
LC4	2	Community activity involvement; access to local information	Survey	Convenience	5	72	Limited awareness of community activities/that activities are community led; barriers to taking part; reliance on word-of-mouth for information.	Context; mechanism of change
LC1	3	Social connection during ‘lockdown’; Access, use, preferences around digital	Structured interview	Random	7	63 (60% under 39 years)	People feel connected enough; widespread access to smartphones and social media; not using email/video calls; mixed access to online services; widespread access to media from other countries; suggestions for a community TV channel.	Context; mechanism of change
LC2	3	Social connectedness and access to support/services during ‘lockdown’	Structured interview/survey	Convenience	7	22 (17 female, 5 male)	Isolating and negative experience; technology useful for maintaining contact but no substitute for in-person; widespread access to IT equipment and social media; limited use of video calls; older people more isolated; digital access requires skills/knowledge; training/support needed.	Context; mechanism of change
LC5	3	Experience of ‘lockdown’; social connectedness and access to support/services during ‘lockdown’; access, use, preferences around digital	Structured interview/survey	Convenience	8	76	Range of negative experiences through lockdown; Most personal circumstances unchanged; availability and quality of support largely unchanged; older people more frequently digitally excluded.	Context; mechanism of change
LC6	3	Social connectedness and access to support/services during ‘lockdown’	Structured interview/survey	Convenience	7	10	Isolating and challenging experience; generally enough access to digital services/support; lack of skills and confidence cause of digital exclusion.	Context; mechanism of change
LC7	3	Experiences of ‘gatekeepers’	Structured interview/survey	Convenience	5	10	Range of gatekeepers; some supportive, some challenging; range of barriers to collaboration; strategies for overcoming barriers.	Context; mechanism of change
LP1	1	Perceptions of the neighbourhood	Semi-structured interviews	Convenience	4	Unclear	People like living in the area; strong local bonds; newcomers take time to integrate; limited local resources; LP project beneficial for participants; range of barriers to taking part.	Context; mechanism of change
LP2	1	Accessibility of the beach/seafront	Mixed-methods – Semi-structured interviews + Feedback at community events + Desk research + photovoice	Convenience	5	Unclear number of interviews. Feedback from over 80 disabled people	Beach/seafront generally inaccessible; inadequate physical infrastructure; negative impacts individuals; multiple potential benefits of improved assess.	Context
LP3	1	Community activity involvement	Mixed-method – Survey + semi-structured interview	Convenience	Unclear	73 survey, 16 interviews	Multiple barriers to participation; complex and dependent on circumstances; more opportunities needed but limited community resources.	Context; mechanism of change
LP4	1	Young resident’s experience of the area	Photovoice	n/a	10 (young people)	10	Plentiful local assets; anti-social behaviour; lack of accessible facilities/places for young people; new youth activities are good; concerns about new housing developments.	Context
LP5	1	Community activity involvement	Semi-structured interviews	Convenience	7	35	Desire for more community activities; clearer information about what’s happening; multiple barriers to taking part.	Context; mechanism of change

**Table 2:** Local people and local conversations peer research process

1*. Peer researchers identified –* Peer researchers recruited by the VCS organisation supporting the programmes. Peer researchers were offered compensation for their contribution in the form of high-street vouchers.	
2. *Peer researcher training workshop –* Peer researchers attended training facilitated by an evaluation team member. Potential research topics, specific research questions/aims, and methodology were discussed and agreed. Peer researchers were advised how to conduct research in an ethical and safe way. Training was planned to last two days but was very often condensed into less than one day to suit peer researchers’ availability.	
3. *Preparation –* The supporting evaluation team member prepared necessary data collection materials (e.g. survey/questionnaire, consent forms) and distributed to peer researchers to use.	
4. *Data collection –* Between four and six weeks was allowed for peer researchers to collect data.	
*5. Data analysis workshop –* A second workshop for peer researchers and supporting evaluation team members to collaboratively analyse the data collected – discussing emergent findings and identifying and agreeing key themes. For quantitative data, some preliminary analysis (e.g. descriptive statistics) was done prior to the workshop by the supporting evaluation team member for the peer researchers to discuss.	
6. *Report writing –* A peer research report was drafted by the supporting evaluation team member and shared with peer researchers for comments/feedback. Amendments were made, and the report signed off by peer researchers.	
7. *Dissemination and integration into wider evaluation –* Peer research sites were given a copy of their report. Reports included in the overall evaluation as a secondary data source.	

Evaluation of both programmes was guided by specific research questions based on a Programme Theory of Change (ToC) [7]. Peer research was one data collection stream in each programme, along with longitudinal qualitative case studies, repeated cross-sectional surveys, process evaluation, and self-evaluation in each case study site. South, Button [7] describe how the overall evaluation methodology accounted for the complexity of the LP intervention. This paper considers whether peer research contributed to the LC and LP evaluations in the way that was planned in the evaluation methodology, namely: to build evaluation skills within participating projects [13], increase the reach of data collection [12], and explore issues that may not be accessible to the evaluation team [15].

## Results

Table 1 summarises key points about the eighteen peer research projects, which are described below in terms of building evaluation skills, the reach of data collection, and issues explored through peer research.

### Building (evaluation) skills and capacity

Peer researcher’ reflections suggest that undertaking peer research helped to build the evaluation and research skills within individual LC and LP projects as well as providing data on other outcomes related to the ToC.

Peer researchers described gaining research skills through participation in peer research. Peer-research also built research capacity within projects. In LC3–1, LC5–3, and LC7–3, projects reported feeling encouraged to do more research after taking part in peer-research. In LC2–3, the community organisation leading the project had commissioned external research that involves peer research, based on their experience during the LC programme.

Peer research also appeared to facilitate some of the LC and LP programmes’ intended shorter term changes. Peer-researchers reported gaining confidence, aspirations, and new interpersonal and technical skills through being involved in the peer-research projects. In LC-1, for example, young people were trained by a professional filmmaker on how to use digital cameras, take photographs, and organise an exhibition. Peer researchers said they generally enjoyed coming together to design and carry out research. Peer-researchers in LC5–3 and LC7–3 stated that doing peer-research was purposeful and engaging. In two projects (LC4–1, LC2–2), gains included more knowledge of their local areas and understanding the experiences of residents which would help inform their project activities.

Some peer-researchers felt unprepared for the activity. This can be attributed to insufficient time being spent to develop peer-researchers’ skills prior to commencing projects. For example, peer-research training was condensed from the planned two sessions into one session due to scheduling issues. In phase 3 of LC, co-analysis workshops were not able to take place due to Covid-19 lockdown restrictions. Other peer researchers expressed frustration at the slow pace of peer-research activities (LC6–3) and a lack of change that occurred as a result of peer-research (LC1–3).

### The reach of data collection

The ‘reach’ of peer research projects concerns the numbers and breadth of both the peer researchers who were involved in each project and the people who contributed as respondents in data collection. Numbers of peer researchers per project ranged from three to ten, with an average of six. They were all local residents and were typically already heavily involved in their respective LP or LC projects as members of steering/advisory groups. Peer researchers were frequently already contributing to the overall evaluation as case study participants via their active roles in LC and LP projects. Demographic information about peer researchers (e.g. age, ethnicity) was not routinely recorded as part of the process, although peer researchers in three projects (LC1–1, LC7–3, and LP4) were young people and in two projects (LC2–3 and LC2–3) peer researchers were all female.

In terms of reach, all but one (LC1–3) peer research projects utilised convenience sampling techniques, such as interviewing friends and neighbours or conducting surveys where local residents already congregated (e.g. community centres). In total, peer research projects engaged 687 people as respondents, including 248 in qualitative methods (ranging from five to thirty-five across individual projects) and 439 survey respondents (ranging from ten to seventy-six across individual projects). Respondents were mostly local residents. One project (LC4–1) also engaged managers of local community assets. Another (LP2–1) was focused on a community of experience rather than one of geography (as was common in most LC and LP projects) and engaged both community members (people with a disability, their families/carers) and non-members (e.g. ‘seafront traders’). Respondents were also mostly people not already actively involved in LC or LP projects or their respective evaluations, indicating the wider reach of the peer research. Demographic information about respondents is inconsistent between projects – it was either not collected or not reported in project reports. Peer researchers in LC7–3, for example, felt very strongly that collecting demographic information was an invasion of privacy and that respondents would feel uncomfortable giving that information. Reasons why it was not collected in other projects is not clear.

### Issues explored

The eighteen peer research projects investigated fourteen separate topics (see Table 1). The most researched topics were around residents’ involvement in community activities (LC3–1, LC3–2, LC5–2, LC4–2, LP3, LP5), residents’ experience of Covid-19 ‘lockdown’ (LC1–3, LC2–3, LC5–3, LC6–3), and the experience of particular groups of residents living in the area (LC1–1, LC2–1, LP4). Other research topics reflected more specific local issues, such as perceptions of proposed estate regeneration (LC3–1), the value of a foodbank (LC5–2), or accessibility to the beach/seafront for people with disabilities (LP2–1).

Mapped against the LC and LP programmes’ ToC, all eighteen projects revealed information about the context in which LC and LP projects occurred. This included things like local housing conditions (LC1–1, LC3–1), local feelings of connectedness (LC1–1, LC2–2, LP2), availability of local resources and community assets (LP1–1, LP3, 1, LC4–1, LP4–1), and mental health stigma in communities (LC2–1). Peer research undertaken during LC phase three provided insight into the local ‘lockdown’ experience (LC1–3, LC2–3, LC5–3, LC6–3). Twelve peer research projects also produced information that could relate to LP and LC ‘mechanisms of change’ – the things LC and LP projects are doing to create change in local areas. Very often this was about barriers and enabling factors to participation in LC and LP project activities and community activities in general (LC3–1, LC3–2, LC5–2, LC4–2, LP3, LP5). Other projects provided information about residents’ previous or other experiences of community involvement and collective action (LC4–1, LC7–3), which provided insight to inform project action. Insights around digital exclusion/inclusion during Covid-19 (LC1–3, LC2–3, LC5–3, LC6–3) could also inform future mechanisms of change.

In terms of how topics were explored, the most frequently used research method (*n* = 8) was surveys, either delivered online, in written format, or over the telephone or face-to-face as a structured interview. Other methods used were semi-structured interviews (*n* = 4) and photovoice [24] (*n* = 3). Three projects used mixed-methods: semi-structured interviews and a survey (*n* = 2), and semi-structured interviews, event feedback, desk research and photovoice (*n* = 1). The research methods used in peer research were very often tailored to residents’ characteristics and needs. Semi-structured interviews were carried out in multiple languages (LC2–1, LC2–2), using photovoice helped engage young people (LC1–1, LP4–1), and surveys were carried out in locations where residents already gathered rather than just being online or via post (LC2–1, LC5–2, LC3–2, LC4–2).

## Discussion

This paper has examined the contribution of peer research to two complex public health intervention evaluations in terms of building evaluation skills within participating projects, increasing the reach of data collection, and exploring issues that may not be accessible to the evaluation team. These three aspects are discussed in turn but with recognition that they overlap and interact.

Firstly, in terms of building evaluation skills, peer research allowed individual peer researchers to learn new research and evaluation skills and for projects to increase their research capacity [13, 14]. This is particularly salient in the context of community empowerment interventions. Including peer research (or other participatory methods) as part of an evaluation of a community empowerment programme can support community empowerment itself, enabling people to come together and engage in dialogue, decision making and action, and, through doing so, increase components of collective control and improve local social determinants of health and wellbeing. Community members gain influence over what constitutes knowledge (and that their lived experiences are a legitimate source of knowledge/evidence) and how that knowledge is produced (via the research questions/agenda they set). This reflects findings in wider literature that bringing stakeholders together provides opportunities for them to learn from each other and from research so that they can act [17, 25]. Peer research also produced a number of shorter-term programme outcomes, including for peer researchers themselves (e.g. confidence, skills, enjoyment, sense of purpose), for LC and LP projects (e.g. increased capacity, gaining new insights into communities, increased capacity to influence local decision makers), and their respective communities (e.g. stimulating social interaction). Again, this reflects findings in the wider literature that participation in research can directly benefit individuals and their community through learning new skills and increased capacity [4, 9, 16], which, in turn, can support the sustainability of community-based interventions [16].

Secondly, in terms of increasing the reach of data collection, peer research projects provided additional opportunities for residents to engage in the overall evaluations. At least some of these residents would have been beyond the reach of the external evaluation teams, such as those who did not speak English, and those who were not engaged with the projects. This reflects the broader value of peer research to facilitate the inclusion of seldom heard voices [12]. However, because demographic information was not routinely collected, it is not possible to say for certain how the respondents compare to those in the other data collection methods. Rather than relying on convenience sampling – a common approach in peer research [26] – purposive or representative sampling to recruit people not represented in other data collection may be an effective way of ensuring peer research increases the reach of data collection. However, this may create additional burdens for peer researchers and/or infringe on peer researchers control of projects.

There was a reliance on those already strongly associated with LC and LP projects and that were already engaged in the respective evaluations as case study respondents to be peer researchers. This means that the benefits of peer research for empowering and upskilling participants and communities [13, 14] were limited to a small pool of people. It is perhaps unrealistic to expect people with no connections to community projects to become effective peer researchers. Whilst training was provided in the technical aspects of research, peer researchers each brought with them important a priori knowledge of projects and trust in the external evaluators. A strategy to broaden the pool of peer researchers may be to actively support people who had been respondents to other data collection methods or other rounds of peer research to become peer researchers. Additionally, while peer-researchers were compensated for their time with high-street shopping vouchers [22], other payment methods (e.g. money) may have been preferred and encouraged greater engagement. Although, many learned bodies have guidance about paying members of the public for their involvement in research, such as NIHR [27], navigating appropriate and fair payment still present challenges for both academics and peer researchers [28]. Future projects may consider taking guidance from potential peer-researchers about their preferred mode and having different ways of compensating different individuals.

Thirdly, in terms of exploring issues that may be beyond the reach of the evaluation team,

the topics of the eighteen peer research projects were broader than the aims and objectives of the overall programme evaluations. All provided insight into the context in which projects occurred, while some informed understanding of programme change mechanisms. This demonstrates that peer research can contribute to an understanding of the context and ecology in which interventions occur [5], enriching interpretation of the mechanisms and processes occurring within programmes [7, 10]. Peer research unpacked community-level processes and perspectives that help explain the interaction of the intervention in context and that have traditionally been insufficiently taken account of in evaluations [8, 21]. The unpredictability of peer research [19] means a potential danger is peer research topics being too removed from the aims and objectives of the evaluation to become irrelevant. Evaluators could mitigate any risk by taking more control of peer research topics, although this would undermine the strength of peer research being led and controlled by people with lived experience. This illustrates the inherent challenge of peer research of getting the balance right between empowering participants and ensuring rigour [20].

Other risks associated with peer research concern data quality and validity [18]. These were mitigated here through training and ongoing support for peer researchers, strategies that have been demonstrated successfully elsewhere [18, 26]. Allowing peer researchers autonomy to adapt data collection methods based on their knowledge of respondents’ needs and preferences also supported validity. Peer researchers generally found the training and support available useful, although some said they felt unprepared. This is perhaps not surprising given that the training was very often condensed to less than one day, allowing little time to ensure all peer researchers sufficiently understood all aspects of the research process. Potentially insufficient ethics and data protection training raises questions about the safety of peer-research projects. However, ongoing support and supervision by a member of the evaluation team helped to mitigate this risk. Further training and support would always be beneficial, although this must be balanced with demands from other aspects of evaluations.

Supporting peer researchers to do co-analysis with the evaluation team was stopped due to Covid-19. The impact of this on individual peer research projects and on evaluations is unknown but it was a necessary pragmatic decision. Likewise, for practical reasons (e.g. researcher and evaluation team capacity, project timelines) peer researchers’ involvement in producing outputs from their projects was limited to commenting on/editing reports drafted by evaluation team members and having final sign-off on completed reports. Whilst this may appear contrary to the principles of peer research, an alternative view is that participation is a continuum, not an absolute [29], and that peer researchers had choices over the extent of their involvement within the confines of this project. Planning additional resources to support fuller participation during this phase may support deeper engagement with analysis and report writing.

A limitation of this current analysis is that it is partially based on limited feedback from peer researchers and observations made whilst supporting peer researcher projects rather than through a systematic evaluation of the peer research process. Further research to, for example, formally assess peer researcher skill acquisition or to compare the composition of respondents to peer research and other data collection methods within evaluations would be useful. While the breadth of the sample of peer research projects across two national community empowerment programmes made it possible to draw out common themes about the contribution of peer research, findings are not generalisable. Further evaluation of peer research in different contexts is merited to examine the transferability and relevance of these themes to other complex public health intervention evaluations.

## Conclusion

This paper has shown that including peer research as part of complex public health intervention evaluations can help uncover important contextual and ecological details beyond the reach of more traditional evaluation data collection, supporting expectations that public health decision makers draw on the best available evidence [2, 30]. Including peer research fits with a growing focus on patient and public involvement in health research [31] and offer a means of expanding the role of members of the public [21] into the design, delivery, and analysis of enquiries. There is distinct value in enabling research that matters to communities. However, balancing increasing community control with managing a complex public health intervention evaluation is challenging and without appropriate planning and resources may necessitate compromising participatory ideals.

## Data Availability

The datasets generated and/or analysed during the current study are not publicly available due to reports that were included in the secondary analysis being co-owned by third party community groups but are available from the corresponding author on reasonable request.

## Abbreviations

LC: Local Conversations (one of the interventions the paper is about)
LP: Local People (one of the interventions the paper is about)
ToC: Theory of Change
VCS: Voluntary and community sector

## References

[1] Petticrew M (2011). When are complex interventions ‘complex’? When are simple interventions ‘simple’?. Eur J Pub Health.

[2] Cyr PR, Jain V, Chalkidou K, Ottersen T, Gopinathan U (2021). Evaluations of public health interventions produced by health technology assessment agencies: a mapping review and analysis by type and evidence content. Health Policy Open.

[3] Minary L, Trompette J, Kivits J, Cambon L, Tarquinio C, Alla F (2019). Which design to evaluate complex interventions? Toward a methodological framework through a systematic review. BMC Med Res Methodol.

[4] Sebastião E, Gálvez PA, Bobitt J, Adamson BC, Schwingel A (2016). Visual and participatory research techniques: photo-elicitation and its potential to better inform public health about physical activity and eating behavior in underserved populations. J Public Health.

[5] Trickett EJ, Beehler S, Deutsch C, Green LW, Hawe P, McLeroy K (2011). Advancing the science of community-level interventions. Am J Public Health.

[6] Orton L, Ponsford R, Egan M, Halliday E, Whitehead M, Popay J (2019). Capturing complexity in the evaluation of a major area-based initiative in community empowerment: what can a multi-site, multi team, ethnographic approach offer?. Anthropol Med.

[7] South J, Button D, Quick A, Bagnall A-M, Trigwell J, Woodward J (2020). Complexity and community context: learning from the evaluation design of a national community empowerment programme. Int J Environ Res Public Health.

[8] Hanson S, Jones A (2017). Missed opportunities in the evaluation of public health interventions: a case study of physical activity programmes. BMC Public Health.

[9] South J, Phillips G (2014). Evaluating community engagement as part of the public health system. J Epidemiol Community Health.

[10] Orton L, Halliday E, Collins M, Egan M, Lewis S, Ponsford R (2017). Putting context Centre stage: evidence from a systems evaluation of an area based empowerment initiative in England. Crit Public Health.

[11] People's Health Trust. Local Conversations. 2021. Available from: https://www.peopleshealthtrust.org.uk/local-conversations.

[12] Vaughn LM, Whetstone C, Boards A, Busch MD, Magnusson M, Määttä S (2018). Partnering with insiders: a review of peer models across community-engaged research, education and social care. Health Soc Care Commun.

[13] Guta A, Flicker S, Roche B (2013). Governing through community allegiance: a qualitative examination of peer research in community-based participatory research. Crit Public Health.

[14] Porter G (2016). Reflections on co-investigation through peer research with young people and older people in sub-Saharan Africa. Qual Res.

[15] Green J, South J (2006). Evaluation.

[16] Wallerstein N, Duran B (2010). Community-based participatory research contributions to intervention research: the intersection of science and practice to improve health equity. Am J Public Health.

[17] Popay J, Whitehead M, Ponsford R, Egan M, Mead R (2020). Power, control, communities and health inequalities I: theories, concepts and analytical frameworks. Health Promot Int.

[18] Lushey CJ, Munro ER (2015). Participatory peer research methodology: an effective method for obtaining young people’s perspectives on transitions from care to adulthood?. Qual Soc Work.

[19] Kavanagh A, Daly J, Jolley D (2002). Research methods, evidence and public health. Aust N Z J Public Health.

[20] Cleaver F, Cooke B, Kothari U (2001). Institutions, agency and the limitations of participatory approaches to development. Participation: the new tyranny?.

[21] Harris J, Croot L, Thompson J, Springett J (2016). How stakeholder participation can contribute to systematic reviews of complex interventions. J Epidemiol Community Health.

[22] Terry L, Cardwell V (2016). Refreshing perspectives: exploring the application of peer research with populations facing severe and multiple disadvantage.

[23] Yin RK (2009). Case study research: design and methods.

[24] Wang C, Burris MA (1997). Photovoice: concept, methodology, and use for participatory needs assessment. Health Educ Behav.

[25] McGill E, Er V, Penney T, Egan M, White M, Meier P (2021). Evaluation of public health interventions from a complex systems perspective: a research methods review. Soc Sci Med.

[26] Woodall J, Cross R, Kinsella K, Bunyan A-M (2019). Using peer research processes to understand strategies to support those with severe, multiple and complex health needs. Health Educ J.

[27] National Institute of Health Research. Payment guidance for researchers and professionals. 2022. Available from: https://www.nihr.ac.uk/documents/payment-guidance-for-researchers-and-professionals/27392.

[28] Cheetham M, Atkinson P, Gibson M, Katikireddi S, Moffatt S, Morris S (2022). Exploring the mental health effects of universal credit: a journey of co-production. Perspect Public Health.

[29] Southby K (2017). Reflecting on (the challenge of) conducting participatory research as a research-degree student. Res All.

[30] Achana F, Hubbard S, Sutton A, Kendrick D, Cooper N (2014). An exploration of synthesis methods in public health evaluations of interventions concludes that the use of modern statistical methods would be beneficial. J Clin Epidemiol.

[31] Pelletier CA, Pousette A, Ward K, Fox G (2020). Exploring the perspectives of community members as research partners in rural and remote areas. Res Involv Engagem.

